# Betrayed gut instinct: Ekbom’s delusion in schizophrenia

**DOI:** 10.1192/j.eurpsy.2026.10513

**Published:** 2026-07-31

**Authors:** F. G. Sousa, M. Vasconcelos, M. Birsanu, C. G. Pereira, D. Mota

**Affiliations:** Centro de Responsabilidade Integrado de Psiquiatria da Unidade Local de Saúde de Coimbra, Coimbra, Portugal

## Abstract

**Introduction:**

Delusional parasitosis, also known as Ekbom’s syndrome, is a rare and strikingly embodied psychotic manifestation, wherein the lived body becomes the stage for persecution and invasion. Though uncommon, it offers a privileged window into the phenomenology of schizophrenia, where delusion, hallucination and the dissolution of self-boundaries intertwine.

**Objectives:**

To describe a case of a nearly decade-long untreated schizophrenia with prominent Ekbom-type delusion, illustrating its rarity, diagnostic complexity and the interplay of factors that shape prognosis and therapeutic engagement.

**Methods:**

Extensive psychiatric evaluation and phenomenological assessment were undertaken, allied with collateral information from medical records, as well as the patient’s autobiographical writings. Complementary tests were conducted to exclude significant organic pathology.

**Results:**

The patient presented with a systematized delusional framework, centered on externally controlled parasites perceived as moving through the brain, arms, abdomen and genitals, with a Koro-like component. Somatic, persecutory, mystico-religious delusions, phenomena of passivity, and multimodal hallucinations completed the clinical picture. The course revealed mixed prognostic indicators; however, optimized treatment in an Advanced Care Unit for Treatment-Resistant Schizophrenia (UCAERe-T) enabled the establishment of a therapeutic alliance and consolidation of insight.

**Conclusions:**

This case underscores the clinical rarity and phenomenological richness of Ekbom’s delusion within schizophrenia. Beyond its narrative vividness, the case exemplifies how multimodal interventions integrating therapeutic alliance, pharmacotherapy, psychotherapy and even specialized units like UCAERe-T can play a decisive role in re-establishing therapeutic trust, fostering insight and offering a renewed horizon of care and better prognosis in treatment-resistant schizophrenia.

**Disclosure of Interest:**

None Declared

